# Inducible HIV-1 Reservoir Quantification: Clinical Relevance, Applications and Advancements of TILDA

**DOI:** 10.3389/fmicb.2021.686690

**Published:** 2021-06-15

**Authors:** Cynthia Lungu, Riddhima Banga, Rob A. Gruters, Francesco A. Procopio

**Affiliations:** ^1^Department of Viroscience, Erasmus University Medical Center, Rotterdam, Netherlands; ^2^Department of Immunology and Allergy, Lausanne University Hospital, Lausanne, Switzerland

**Keywords:** HIV-1 latency, HIV-1 cure, reservoir quantification, inducible reservoir, TILDA

## Abstract

The presence of a stable HIV-1 reservoir persisting over time despite effective antiretroviral suppression therapy precludes a cure for HIV-1. Characterizing and quantifying this residual reservoir is considered an essential prerequisite to develop and validate curative strategies. However, a sensitive, reproducible, cost-effective, and easily executable test is still needed. The quantitative viral outgrowth assay is considered the gold standard approach to quantify the reservoir in HIV-1-infected patients on suppressive ART, but it has several limitations. An alternative method to quantify the viral reservoir following the reactivation of latent HIV-1 provirus detects multiply-spliced *tat/rev* RNA (msRNA) molecules by real-time PCR [*tat/rev* induced limiting dilution assay (TILDA)]. This article provides a perspective overview of the clinical relevance, various applications, recent advancements of TILDA, and how the assay has contributed to our understanding of the HIV-1 reservoir.

## Introduction

Over the past decade, multiple interventions to reduce or eliminate the latent HIV-1 reservoir have been proposed. A prominent strategy is to pharmacologically reactivate latently infected cells through so called latency reversal agents (LRAs) in conjunction with immune-modulating interventions to enhance extrinsic immune-based clearance or induce intrinsic apoptosis of reactivated cells (Kim et al., 2018; Abner and Jordan, 2019; Collins et al., 2020). Other eradication strategies include genome editing of target molecules such as the HIV co-receptor *CCR5* (Almeida and Matos, 2019) or on the HIV-1 genome itself using CRISPR/Cas9 (Zhu et al., 2015) (reviewed in Wang et al., 2018) or Zinc finger nucleases (Qu et al., 2013). “Block and lock” strategies using pharmacological compounds (Ahlenstiel et al., 2015; Mousseau et al., 2015) or *via* therapeutic vaccination using broadly neutralizing monoclonal anti-HIV-1 antibodies (bNAbs) (Mendoza et al., 2018) to permanently silence the provirus have also been proposed. The advent of these strategies has prompted the development of several methods to quantify the HIV-1 reservoir and validate the effectiveness of potential cure interventions. However, existing cure strategies require further optimization and new assays with higher sensitivity, accuracy, precision, and reproducibility (Falcinelli et al., 2019) are urgently needed. A panel of latent HIV-1 reservoir assays have been recommended for use in HIV cure intervention trials (reviewed in Abdel-Mohsen et al., 2020). The quantitative viral outgrowth assay (QVOA), currently considered the reference assay for quantifying the replication-competent HIV-1 reservoir (Eriksson et al., 2013), quantifies the number of resting CD4^+^ T cells releasing infectious virus after *in vitro* stimulation and subsequent co-culture with feeder cells (Siliciano and Siliciano, 2005; Laird et al., 2016). However, the widespread use of QVOA in clinical trial settings is precluded by several factors related to the complexity of the assay {large blood draws, high variability [recently evaluated in the RAVEN studies (Rosenbloom et al., 2019; Stone et al., 2020)], labor intensity, and execution time (14–21 days)}. Moreover, QVOA underestimates the size of the reservoir and has a low dynamic range (Ho et al., 2013). Several efforts to improve QVOA (sensitivity and dynamic range, assay turn-around time, and overall costs) have been implemented (Passaes et al., 2017; Massanella et al., 2018; Stuelke et al., 2020; Zhang et al., 2020). A general concern, however, is that QVOA protocol modifications further complicate assay standardization (Stone et al., 2020). Alternatively, real-time or digital PCR-based methods, which are better suited for large clinical trials, have widely been used to detect HIV-1 DNA in infected cells from peripheral blood and lymphoid tissue cells (Vandergeeten et al., 2014; Alidjinou et al., 2015; Avettand-Fenoel et al., 2016; Anderson and Maldarelli, 2018). Although these methods are relatively easy to perform, fast, and cost-effective, they overestimate the reservoir size because most integrated HIV-1 genomes are not replication-competent (Ho et al., 2013). Advanced HIV-1 DNA quantification methods such as the intact proviral DNA assay (IPDA) (Bruner et al., 2019) and Q4PCR (Gaebler et al., 2019), distinguish intact proviruses from defective ones thereby providing a better resolution for studying the dynamics of defective and intact HIV-1 proviral DNA (Falcinelli et al., 2020; Peluso et al., 2020; Simonetti et al., 2020). However, proviral intactness does not guarantee virion production (Ho et al., 2013; Siliciano and Siliciano, 2021), and the fraction of intact proviruses that can be induced to produce virions cannot be determined by these assays, which is a critical limitation given that many intact proviruses exhibit low inducibility (Ho et al., 2013; Siliciano and Siliciano, 2021). In long-term ART-suppressed people living with HIV (PLWH), intact HIV-1 proviruses are preferentially integrated in an opposite orientation to host genes, in relative proximity or increased distance from active transcriptional start sites and accessible chromatin regions (Einkauf et al., 2019). The transcription-competent (inducible) HIV-1 reservoir can be quantified by several methods (Bullen et al., 2014; Cillo et al., 2014; Yucha et al., 2017; Massanella et al., 2018) including an approach developed by Procopio et al. (2015), which detects multiply-spliced *tat/rev* RNA (msRNA) molecules by real-time PCR [*tat/rev* induced limiting dilution assay (TILDA)], and reduces the likelihood of quantifying defective genomes (Procopio et al., 2015; Imamichi et al., 2016; Pollack et al., 2017). The percentage of intact proviruses capable of producing *tat/rev* msRNA transcripts is uncertain but a recent study showed no association between inducible HIV measures (TILDA, p24 SIMOA) and IPDA-intact proviruses but with IPDA-total measures (Papasavvas et al., 2021). TILDA correlates poorly with QVOA (Procopio et al., 2015; Stone et al., 2020), which may be explained by several factors including: technical (inherent assay variation and poor sensitivity); differences in provirus inducibility (Ho et al., 2013; Siliciano and Siliciano, 2021); the biological targets measured as readouts [*tat/rev* msRNA (TILDA) and HIV-1 p24 *gag* RNA or Gag protein (QVOA)] (Massanella et al., 2018); *tat/rev* msRNA is measured after 12–18 h of activation, whereas QVOA measurements are taken after 7–14 days of culture and virus production. TILDA probes total CD4^+^ T cells, whereas QVOA uses sorted resting CD4^+^ T cells. Furthermore, due to post-transcription blocks in RNA processing (Yukl et al., 2018), not all cells producing *tat/rev* msRNA transcripts will yield infectious virus (Hong et al., 2018), and therefore quantifying these cells overestimates the replication-competent HIV-1 reservoir size. Despite these discrepancies and limitations, TILDA is a well-established approach to quantify the inducible HIV-1 reservoir as a proxy for replication competence, and a technically feasible alternative to QVOA for routine execution in large scale HIV cure intervention trials [requires less blood, shorter assay turn-around time (2 days), and medium throughput]. In this article we provide a perspective overview of the clinical relevance, various applications, and recent advancements of TILDA, and how the assay has contributed to our understanding of the HIV-1 reservoir.

## Principle of the *tat/rev* Induced Limiting Dilution Assay

The fundamental steps in performing TILDA as published by Procopio et al. (2015) include: (i) isolation of CD4^+^ T cells from 10 mL blood collected from HIV-1-infected individuals; (ii) *in vitro* stimulation for 12 h with 100 ng/mL phorbol 12-myristate 13-acetate (PMA) and 1 μg/mL ionomycin to induce production of *tat/rev* msRNA; (iii) distribution of cells in a limiting dilution scheme (24 replicates of 1.8 × 10^4^ to 1.0 × 10^3^ cells per well) in a 96-well plate containing a one-step reverse transcription PCR (RT-PCR) master mix, which bypasses RNA extraction, allowing simultaneous RT and pre-amplification of *tat/rev* msRNA; (iv) quantification of *tat/rev* msRNA using a diluted fraction of the pre-amplification product; (v) estimation of the frequency of *tat/rev* msRNA^+^ cells using maximum likelihood method (Hu and Smyth, 2009). TILDA has a limit of detection of ∼1 cell expressing *tat/rev* msRNA per million CD4^+^ T cells, a coefficient of variation of ∼20% (Procopio et al., 2015; Lungu et al., 2020), and correlates with various assays to measure latency, reactivation and reservoir (total and integrated HIV DNA, p24 SIMOA, and HIV FISH-Flow) (Procopio et al., 2015; Pardons et al., 2019; Papasavvas et al., 2021). In-depth comparisons of these assays have been reviewed elsewhere (Eriksson et al., 2013; Siliciano and Siliciano, 2018; Falcinelli et al., 2019; Abdel-Mohsen et al., 2020).

## Clinical Relevance of Inducible HIV-1 Reservoir Quantification by TILDA

In ART-suppressed PLWH, the median frequency of latently infected CD4^+^ T cells estimated by TILDA is ∼24 per million CD4^+^ T cells (Procopio et al., 2015; Pardons et al., 2019; Lungu et al., 2020), 48 times larger than the frequency measured with QVOA. Nevertheless, the median frequency of intact, non-induced proviruses is reported to be 60-fold higher than the frequency of induced proviruses detected in QVOA (Ho et al., 2013). The chromosomal integration site is a critical factor determining provirus inducibility and there is recent evidence that long-term ART-suppressed PLWH harbor intact proviruses with features of deeper viral latency (Battivelli et al., 2018; Einkauf et al., 2019). TILDA readouts therefore serve as intermediate, surrogate measures for the frequency of latently infected cells harboring replication-competent HIV-1. Procopio et al. (2015) demonstrated that the size of the inducible HIV-1 reservoir was smaller in subjects who initiated ART during acute HIV infection (AHI) when compared to those who began therapy at a later stage of the disease (median frequencies of 11 and 29 cells per million CD4^+^ T cells in acute and chronic infection, respectively). This trend has also been shown in other studies using QVOA and HIV-1 DNA (total and integrated) (Buzon et al., 2014; Ananworanich and Mellors, 2015). Notably, the frequency of cells with inducible msRNA in most samples obtained from individuals who initiated ART during Fiebig stages I to III is even lower (Leyre et al., 2020), nearing or below the TILDA limit of detection (Henrich et al., 2017; Colby et al., 2018; Leyre et al., 2020). Comparing TILDA estimates before and after stimulation of CD4^+^ T cells obtained from ART-naïve individuals, most infected cells (81.4%) were latently infected and produced msRNA only after stimulation. In contrast, merely 18.6% of the infected cells spontaneously produced msRNA (Procopio et al., 2015). Therefore, similarly to ART-suppressed PLWH, ART-naïve individuals harbor a large pool of latently infected CD4^+^ T cells. Moreover, the frequency of latently infected CD4^+^ T cells in ART-naïve individuals positively correlated with the duration of HIV-1 infection, which suggests that the size of the latent and inducible reservoir increases over time in ART-naïve individuals as a result of continuous seeding of the HIV-1 reservoir (Procopio et al., 2015). A recent study reported a correlation between the inducible reservoir measured by TILDA and the CD4/CD8 ratio (Bertoldi et al., 2020), an important clinical parameter reflecting the immune system’s functionality and linked to reservoir size (Chun et al., 2002; Lu et al., 2015).

## Application of TILDA in HIV-1 Cure Interventions

### Evaluating Latent HIV-1 Reservoir Reactivation and Elimination Using TILDA

Transcriptional activation of HIV-1 provirus from latently infected cells using LRAs and subsequent immune-mediated clearance is viewed as one of the most promising approaches toward HIV-1 eradication. During the last years, multiple LRAs have been tested *in vitro* and *ex vivo*, and in combination with other interventions in clinical trials (reviewed in Stoszko et al., 2019; Zerbato et al., 2019). In two clinical trials evaluating depsipeptide Romidepsin’s latency reversal potential *in vivo*, no substantial changes in HIV-1 reservoir size could be observed by total HIV-1-DNA quantification, QVOA, or TILDA (Sogaard et al., 2015; McMahon et al., 2020), despite detectible HIV-1 viral reactivation measured using standard plasma HIV-1 RNA assays. In another *in vivo* clinical study, the histone deacetylase inhibitor Vorinostat induced a sustained increase of CA unspliced HIV-1 RNA from CD4^+^ T cells from the blood. Still, there was no significant change in plasma HIV-1 RNA or HIV-1 reservoir size measured by TILDA and HIV-1 DNA (total and integrated) (Elliott et al., 2014). These and other studies highlight that while it is possible to disrupt HIV-1 latency in patients on suppressive ART, the LRA interventions so far have not significantly impacted the latent HIV-1 reservoir size or time to viral rebound (reviewed in Zerbato et al., 2019).

All considered, TILDA could be valuable in LRA studies; the initial step of viral reactivation is the early expression of *tat* and *rev* RNAs, the assay’s primary targets. Therefore, TILDA would be an ideal screening tool to validate promising compounds. For instance, TILDA contributed to defining a new class of novel pure phorbol ester-class compounds, isolated from an African medicinal plant (Mukungulu) (Tietjen et al., 2018), with the capacity to promote HIV-1 latency reversal *ex-vivo* in J-Lat cells containing an HIV-1-GFP provirus (Tietjen et al., 2019). TILDA results could also be used to differentiate T cell populations with large and small inducible HIV-1 reservoirs. In multiple studies, TILDA revealed that CD4^+^ T effector memory cells harbor the largest inducible HIV-1 reservoir in peripheral blood when compared to CD4^+^ T central memory cells and CD4^+^ T transcriptional memory cells (Fromentin et al., 2016; Kulpa et al., 2019). This finding suggests the possibility to induce the differentiation toward effector memory cells as a strategy to increase the HIV-1 reservoir susceptibility to LRAs (Hiener et al., 2017; Wonderlich et al., 2019; Kwon et al., 2020; Rabezanahary et al., 2020), and TILDA may be useful for measuring the effects of such novel approaches.

Furthermore, innovative strategies to enhance killing mechanisms in different T cell populations are gaining more attention (reviewed in Kim et al., 2018). Several recent studies have demonstrated the use of pharmacological compounds that selectively induce cell apoptosis in HIV-1-infected cells as a strategy to eliminate these cells (Campbell et al., 2018; Matsuda et al., 2019; Zhang et al., 2019). In a study by Rao et al. (2021), CD4^+^ T cells obtained from ART-suppressed HIV-1-infected individuals were cultured for 5 days in the absence or presence of DDX3 inhibitors. The addition of DDX3 inhibitors resulted in a reduction (∼50%) of the inducible HIV-1 reservoir, determined by quantifying CA HIV-1 RNA, the frequency of cells expressing *tat/rev* msRNA (TILDA), and the frequency of cells positive for *gag* RNA using FISH-flow (Rao et al., 2021).

### Characterizing Latently Infected Cells Using TILDA

The elimination or reduction of HIV-1-infected cells is a priority toward an HIV cure. Unfortunately, there is not a specific biomarker for the identification of infected cells. It is well known that CD4^+^ memory T cells (Chomont et al., 2009) and CD4^+^ T cells with stem cell-like properties (Buzon et al., 2014) in peripheral blood, and follicular helper T (Tfh) cells (PD-1^high^/CXCR5^high^) and memory CD4^+^ T cells (Banga et al., 2016) in lymph nodes are enriched in cells harboring replication-competent HIV-1. These cell populations share multiple co-inhibitory molecules (immune checkpoints, ICs), which down-modulate the immune response and prevent hyper-immune activation. ICs are typically upregulated after T-cell activation, and their overexpression is associated with T-cell exhaustion in cancer and chronic viral infections, including HIV-1 (Day et al., 2006; Petrovas et al., 2006; Trautmann et al., 2006). A recent study identified PD-1, TIGIT, and LAG-3 as ICs, which are positively associated with the frequency of CD4^+^ T cells harboring integrated HIV-1 DNA (Fromentin et al., 2016). Notably, the same populations were threefold more positive for *tat/rev* msRNA when compared to the PD-1^neg^, TIGIT^neg^, and LAG-3^neg^ CD4^+^ T cell populations, suggesting that resting or exhausted cell status favors HIV-1 persistence. Integrated HIV-1 DNA, TILDA, and plasma HIV-1 RNA measurements were also used to characterize the HIV-1 reservoir in another study that aimed to identify molecular signatures that may support the role of Tfh cells as a significant compartment for HIV-1 persistence (Aid et al., 2018). Notably, BCL6 gene expression, which is the master regulator of Tfh cell differentiation and modulator of a series of other transcription factors and their downstream targets, positively correlated with integrated HIV-1 DNA as well as TILDA and plasma viral load (Aid et al., 2018). Multiple studies demonstrate that α4β7-expressing cells represent early targets for HIV-1 and that pre-infection frequencies of α4β7 on circulating CD4^+^ T cells may predict the risk of HIV-1 acquisition and disease progression (Cicala et al., 2009; Kader et al., 2009; Martinelli et al., 2013; Sivro et al., 2018). In a phase I clinical trial to study the impact of anti-α4β7 monoclonal infusion on HIV-1 reservoir size TILDA was used to determine change in inducible reservoir size after treatment. A reduction in msRNA post-treatment was observed in two out of four donors, but the low sample size impaired the test’s statistical significance (Uzzan et al., 2018).

## TILDA Advancements

### Broadening the Detection of *tat/rev* msRNA Beyond HIV-1 Clade B

**FIGURE 1:**
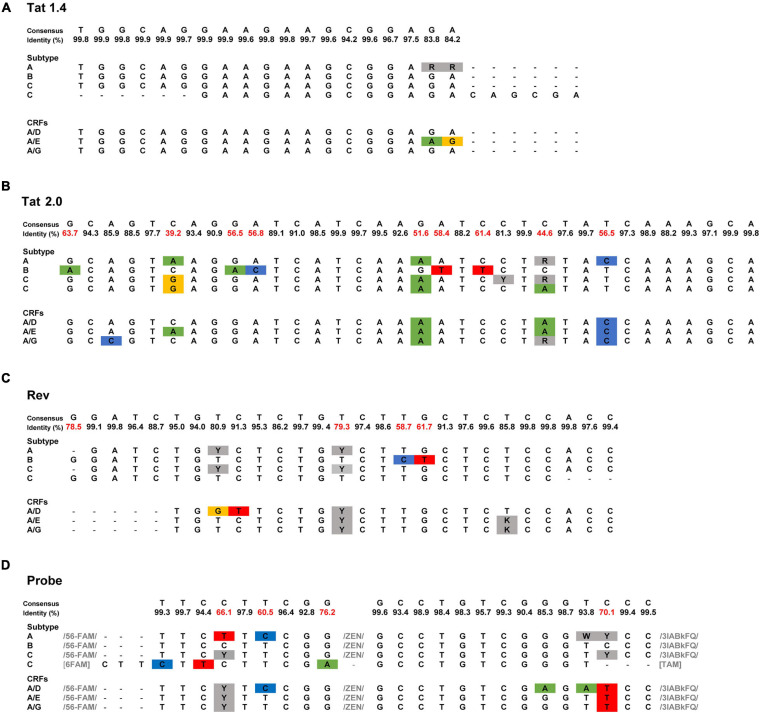
Sequence of TILDA primers and probes specific for different HIV-1 subtypes. **(A–D)** TILDA primer and probe sequences were retrieved from the literature [HIV-1 subtype: A (Dhummakupt et al., 2020), B (Procopio et al., 2015), C (Bertoldi et al., 2020; Dhummakupt et al., 2020), A/E (Colby et al., 2018), A/D, and A/G (Dhummakupt et al., 2020)] and aligned to a consensus sequence generated from an alignment of 2695 HIV-1 sequences downloaded from the Los Alamos HIV-1 sequence database. Nucleotide substitutions are colored blue, green, yellow, and red. Nucleotide positions with degenerate bases are colored gray. The least conserved nucleotide positions (<80% identity) are marked in red.

High sequence diversity in the *tat/rev* region demands the modification of primers and probes to increase TILDA’s specificity for different HIV-1 clades. In its original design, TILDA optimally quantifies the HIV-1 reservoir in individuals infected with clade B (Procopio et al., 2015), the dominant clade in North America and Europe. This primer and probe restriction is a limitation considering that 46.6% of HIV-1 infections worldwide are clade C, the most dominant HIV-1 clade in Africa (Hemelaar et al., 2019). Given this, C-TILDA was recently developed with primers and probes modified specifically for clade C viruses (Bertoldi et al., 2020), which extends the possibility of screening other HIV-1-infected populations and implementing the assay in eradication strategies in regions where clade C is more abundant. In two other studies, primers and probes were designed to efficiently amplify *tat/rev* msRNA for clade A/E, A1, and A/G viruses as well (Colby et al., 2018; Dhummakupt et al., 2020). We aligned the published TILDA primer and probe sequences to a consensus sequence generated from 2695 global HIV-1 sequences downloaded from the Los Alamos HIV-1 sequence database (Figure 1). The highest degree of variability is evident in the Tat 2.0 forward primer in which 9/34 positions have <80% (39.2–63%) identity to consensus. Further studies should validate a generalized approach using combinations of primer and probe sets for these and other circulating HIV-1 subtypes, broadening the detection and increasing the assay’s applicability.

Indeed, non-human primate (NHP) models are critical to test novel therapeutic strategies and better understand the HIV-1 reservoir (Whitney et al., 2014; Nixon et al., 2017). Considering the limited number of cells available, performing QVOA is impractical in NHP studies, therefore Frank et al. (2019) developed a simian version of TILDA (termed SIV/SHIV-1 TILDA) to detect msRNA of SIVmac251/mac239 and SHIV-1_*AD80E*_ viruses, which are widely employed in NHP studies. The SIV/SHIV-1 TILDA could detect msRNA also in the setting of very low/undetectable viremia. Interestingly, in animals that started ART early after infection, the results from reservoir estimation by TILDA correlated with the viral rebound post ART interruption (Frank et al., 2019).

### Improving TILDA Sensitivity

**FIGURE 2:**
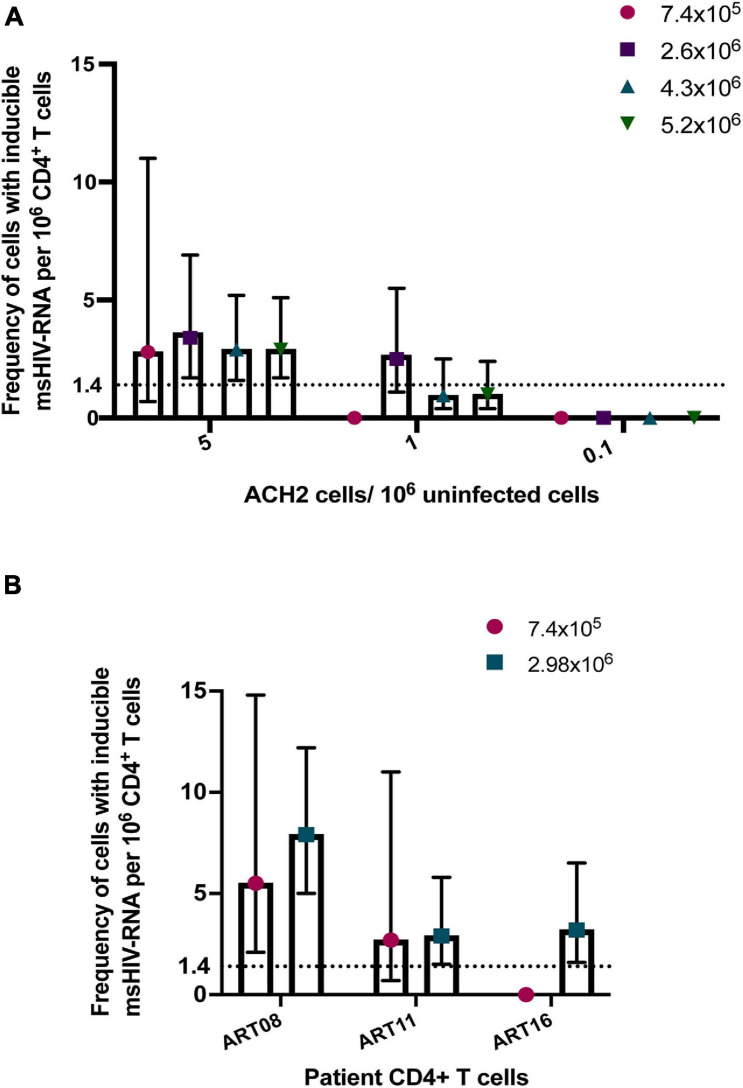
Detection of *tat/rev* msRNA in low reservoir samples. **(A)** Target detection in three low concentrations of ACH-2 cells mixed with one million uninfected CD4^+^ T cells. The total number of cells tested per plate ranged from 7.4 × 10^5^ cells to 5.2 × 10^6^ cells **(B)** CD4^+^ T cells obtained from three ART-suppressed individuals (plasma HIV RNA <20 c/mL) were stimulated with PMA/ionomycin for 12–14 h. The frequency of cells expressing *tat/rev* msRNA was determined using TILDA v2.0 (Lungu et al., 2020) with standard (7.4 × 10^5^ cells) or higher cell inputs (colored symbols). The mean estimates of inducible cells/10^6^ CD4^+^ T cells are plotted in both graphs. The error bars represent the 95% confidence interval upper and lower limits. The horizontal line represents the assay limit of detection (LOD) based on the standard cell input (7.4 × 10^5^ cells).

A major advantage of TILDA is the direct amplification of *tat/rev* msRNA from whole cell lysate. However, the cell lysate complexity itself can reduce the signal to noise ratio impacting overall *tat/rev* msRNA amplification efficiency. To improve msRNA detection, a new TILDA protocol that includes an RNA extraction step was developed, the results of which strongly correlated with those of the original protocol (Pezzi et al., 2017). In a recent study, activating cells with phytohemagglutinin for an extended period (18 h instead of 12 h) enhanced the expression of *tat/rev* msRNA in CD4^+^ T cells isolated from infants living with HIV-1 (Dhummakupt et al., 2020). Several studies have shown that the frequency of cells that could be induced to express HIV-1 msRNA is small in most individuals treated during AHI (Fiebig stages 1-III) (Colby et al., 2018; Leyre et al., 2020). Furthermore, some long-term suppressed patients or elite HIV-1 controllers have significantly lower latent viral reservoirs (Siliciano et al., 2003; Autran et al., 2011; Bertoldi et al., 2020; Galvez et al., 2020). In these cases, reservoir estimation using TILDA is technically challenging. A higher cell input is required to increase the assay’s sensitivity, which can be achieved by increasing the number of replicates through processing multiple assay plates (Procopio et al., 2015). However, processing multiple TILDA plates per sample reduces assay throughput and increases the overall cost. To circumvent these limitations, maximizing the number of cells that can be probed per reaction is essential. In the recently developed C-TILDA (Bertoldi et al., 2020), the authors increased cell input (up to 5.4 × 10^4^ cells per reaction instead of 1.8 × 10^4^) to maximize detection of *tat/rev* msRNA. In another study, which assessed assay robustness and amenability for routine use in clinical studies of the latent reservoir, TILDA was performed using alternative RT-qPCR reagents according to a modified (TILDA v2.0) protocol (Lungu et al., 2020). Particularly, *tat/rev* msRNA pre-amplification reactions were carried out in larger volumes (10 μl instead of 1 μl) to minimize pipetting imprecisions. Furthermore, the pre-amplification products were directly added to the real-time PCR mix to detect msRNA, which improves assay throughput by reducing the number of pipetting steps and minimizes the risk of cross-contamination associated with diluting PCR products (Lungu et al., 2020). In our preliminary assessment of TILDA v2.0 performance, using a higher input number of ACH-2 or HIV-1-infected CD4^+^ T cells per reaction (7.2 × 10^4^ cells to 1.4 × 10^5^ cells), resulting in six to eightfold more cells per assay, improved target detection and precision (narrower 95% confidence intervals) when quantifying small reservoirs (Figure 2). Further studies are necessary to assess TILDA or TILDA v2.0 reproducibility with these higher cell inputs, which require an increased total blood volume of 30–50 mL, and the capacity thereof to quantify inducible reservoirs below the current detection limits. Indeed, such validation studies would reveal more insight into TILDA’s suitability for evaluating HIV-1 reservoir-reduction clinical interventions.

## Conclusion

As we advance toward large, scalable clinical interventions to significantly deplete or permanently restrict inducible HIV-1 reservoirs in well-suppressed PLWH, TILDA stands out as a suitable option for implementation in the aforementioned HIV cure clinical trials. Within 2 days, inducible HIV-1 reservoir measurements can be generated using less than 1 × 10^6^ viable target cells per condition with the possibility to increase cell input [six to eightfold (TILDA v2.0)] without significantly impacting overall assay costs. The assay is also very robust, as demonstrated in multiple studies to test intra- and inter-laboratory reproducibility, which is important for the cross-validation of results in multi-center, multi-laboratory clinical trials.

## Data Availability Statement

The raw data supporting the conclusions of this article will be made available by the authors, without undue reservation.

## Ethics Statement

The studies involving human participants were reviewed and approved by the Erasmus MC Medical Ethics Committee MEC-2016-148 (July 22, 2016). The patients/participants provided their written informed consent to participate in this study.

## Author Contributions

CL and FP conceptualized the perspective article, researched the literature, and wrote the first draft of the manuscript. CL performed the experiments and analyzed the data. All authors read and approved the final manuscript.

## Conflict of Interest

The authors declare that the research was conducted in the absence of any commercial or financial relationships that could be construed as a potential conflict of interest.
